# Global trends of researches on bone metastasis: A bibliometric and visualization study

**DOI:** 10.1097/MD.0000000000028761

**Published:** 2022-02-11

**Authors:** Kai Huang

**Affiliations:** Department of Spinal Orthopaedics, The Second People's Hospital of Changshu, Changshu, China.

**Keywords:** bibliometric analysis, bone metastasis, research trends, visualization

## Abstract

**Background:**

Bone metastasis (BM) has become an important health problem. In recent years, studies on BM are growing rapidly, but there were no bibliometric studies regarding BM. This study aimed to illustrate the overall knowledge structure and development trends of BM.

**Methods:**

Research datasets were acquired from the Web of Science database. The time span was defined as “1980–2020”. VOS viewer and Citespace software was provided to analyze the data and generate visualization knowledge maps. Annual trends of publications, distribution, H-index status, co-authorship status, and research hotspots were analyzed.

**Results:**

Six hundred eighty-two publications met the requirement. USA published most papers (264, 38.7%), and both total citations (13,997) and H-index (57) of USA ranked first. The most productive institution on BM is *Amgen Inc.* (43). *Supportive Care in Cancer* (24) published the most papers on BM. “Safety”, “skeletal related event”, “open label”, “management”, “health”, and “prognosis” are the research hotspots in the recent years.

**Conclusion:**

In this study, we conduct a systematic and comprehensive analysis on the research in BM field. The publication number was rising in recent years stably. USA contributed mostly not only in quality, but also in quantity. *Amgen Inc.* published the largest number of articles, *Supportive Care in Cancer* was the most productive journal related to BM. “Safety”, “skeletal related event”, “open label”, “management”, “health”, and “prognosis” are the research hotspots in recent years. We believe this study can not only show the global research overview in past 40 years but also point the research trend of BM in the future.

## 1 Introduction

The bone is the third most common site of metastasis for a wide range of solid tumors including lung, breast, prostate, colorectal, thyroid, gynecologic, and melanoma, with 70% of metastatic prostate and breast cancer patients harboring bone metastasis (BM).^[1]^ Once the tumor has metastasized to the bone, the death of the patient is significantly increased and cannot be cured.^[2,3]^ In clinical work, with the development of malignant tumor treatment technology, the survival time of many tumor patients has increased significantly, and the incidence of malignant tumor BM has also increased accordingly. Therefore, malignant tumor BM has become one of the hot issues in clinical orthopedics research.

Bibliometrics is the cross-disciplinary science of quantitative analysis of all knowledge carried by mathematical and statistical methods.^[4]^ It has been applied to evaluate citation counts and collaboration in countries, institutions, journals, and authors, and to predict key word trends in the research field.^[5]^ So it plays a great role in evaluating scientific fields.

However, to our knowledge, bibliometric studies concerning the trend of the published literature of articles published in the field of BM has not yet been reported. This study aimed to intuitively show the research framework, overall knowledge structure, and development trends of the field of BM. We hope this study will help scientific scholars better understand the research status and development trends.

## 2 Materials and methods

### 2.1 Search strategy and refined data

The data for this study were collected from the Web of Science (WOS) and its Core Collection. WOS is a most widely accepted and suitable database for the subsequent bibliometric analysis of scientific publications due to its strict evaluation process and the most influential and credible information it could provide.

The literature search was also limited to articles that were published from January 1, 1980 until August 1, 2020 (a span of 40 years). The search terms were integrated as “bone metastasis” OR “bone metastases” AND “solid tumor”. Original articles were included in this study, while letters, editorials, basic research articles, duplicate articles, non-English literatures, and corrections were excluded in our finalize data set. To perfect the research, 2 independent researchers reviewed and evaluated the cited articles, respectively. Any different opinions were discussed until consensus was reached.

### 2.2 Data analysis

All data were extracted and imported into Microsoft Excel 2017. Annual trends of publications, distribution, citation and H-index status, co-authorship status, research hotspots, and co-citation status were analyzed. Chi-square analysis was performed using SPSS version 20.0 (SPSS Inc.). Statistical significance was considered at *P* < .05. VOS viewer (Eck and Waltman) and Citespace (Chaomei Chen) are used to quantify information, visualize co-occurrence networks utilizing various layouts, and create timeline view of the keywords.^[6,7]^

## 3 Results

### 3.1 The current status and annual trend of study

**Figure 1:**
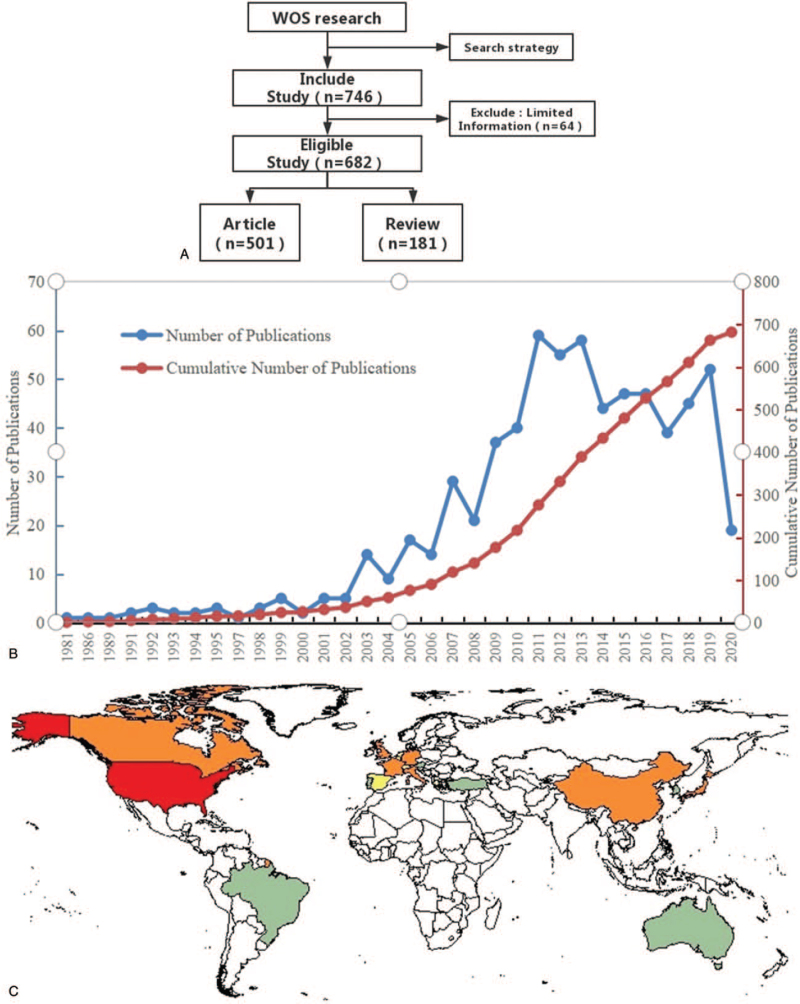
(A) Flow chart; (B) the annual trends of publications; (C) map of worldwide research productivity.

A total of 746 articles on BM were identified in the WOS database as a result of the search. With an additional manual screening according to the inclusion criteria, a total of 682 articles were analyzed finally. Due to the absence of relevant information such as the author, the journal, or years, 64 study with limited information were removed. One hundred eighty-one papers are reviews and 501 papers are articles. The selection flow chart is shown in Figure 1A. The sum number of citations is 23,177 (21,035 without self-citations). The average citation of all the papers is 33.98 times. The H-index of all the publications related to BM is 72.

Only 1 article was published in 1981, and the growth was found in the following year. There was a peak in the number of studies from 2009 to 2014. A total of 59 articles (8.65%) were published in 2011, the highest number in all years, followed by the year 2013 (58, 8.5%) and the year 2012 (206, 8.06%). The results showed that the most efforts and exploration have been made on BM from 2011 to 2013. However, 52 articles were published in 2019, which indicated that research trends on BM are gradually picking up in recent years. Figure 1B plots the annual trends of publications. The world-map distribution shows that most of the publication are from North America, Europe, and Asia (Figure 1C).

### 3.2 The distribution and co-authorship analysis of countries

**Figure 2:**
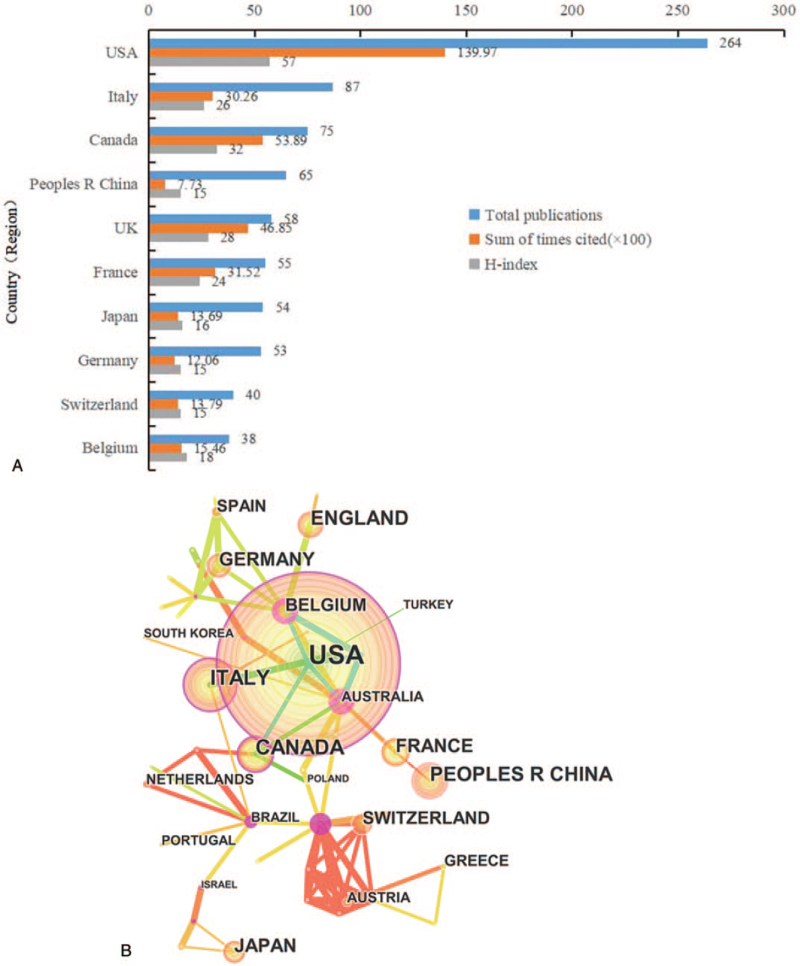
(A) The publication number, H-index, and cited times of the top 10 countries; (B) the countries co-authorship network of publications.

A total of 51 countries contributed to the field of bone metastases research. But the majority of the papers were published in only a few countries. There were 549 papers (80.49%) published in the top 5 countries, and most of the studies were from North America and Europe. The United States published the largest number of articles (264, 38.70%), followed by Italy (87, 12.76%), Canada (75, 10.99%), China (65, 9.53%), and UK (58, 8.50%). H-index is a reliable and authentic parameter for academic evaluation. The United States had the highest H-index (57), followed by Canada (32), UK (28), Italy (26), and China (17; Table 1). The result shows that USA and Italy are the countries with the highest productivity, not only in qualities, but also in quantities. Canada, China, and UK are the other most contributing countries (Fig. 2A). The Citespace viewer software was employed to analyze the network visualization of co-authorship relationship. Only countries with a minimum of 7 articles were included. USA was at the center of research and stayed in closest collaboration with Italy and Canada. Nevertheless, these cooperation between the countries were relatively weak (Fig. 2B).

**Table 1:**
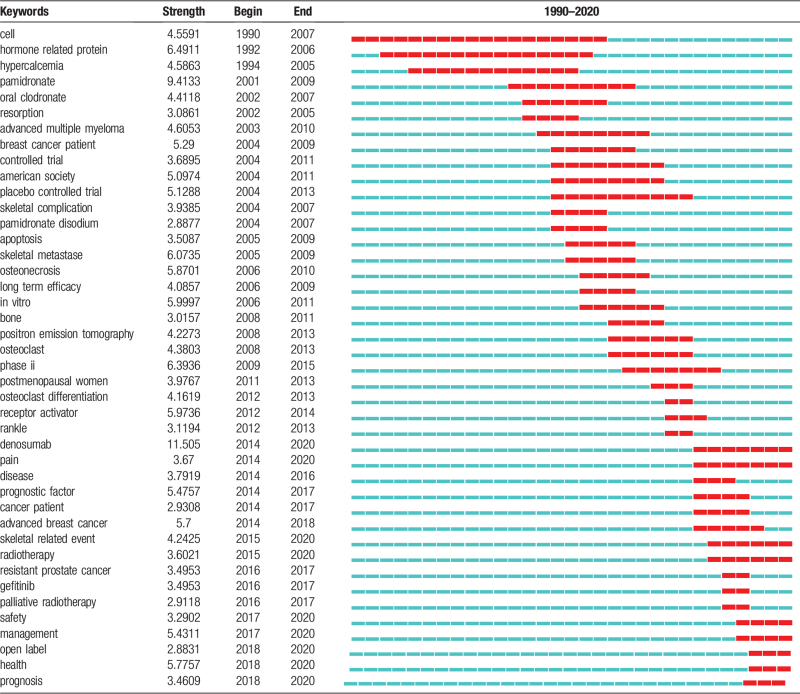
Top 42 keywords with the strongest citation bursts.

### 3.3 The distribution and co-authorship analysis of published journals

**Figure 3:**
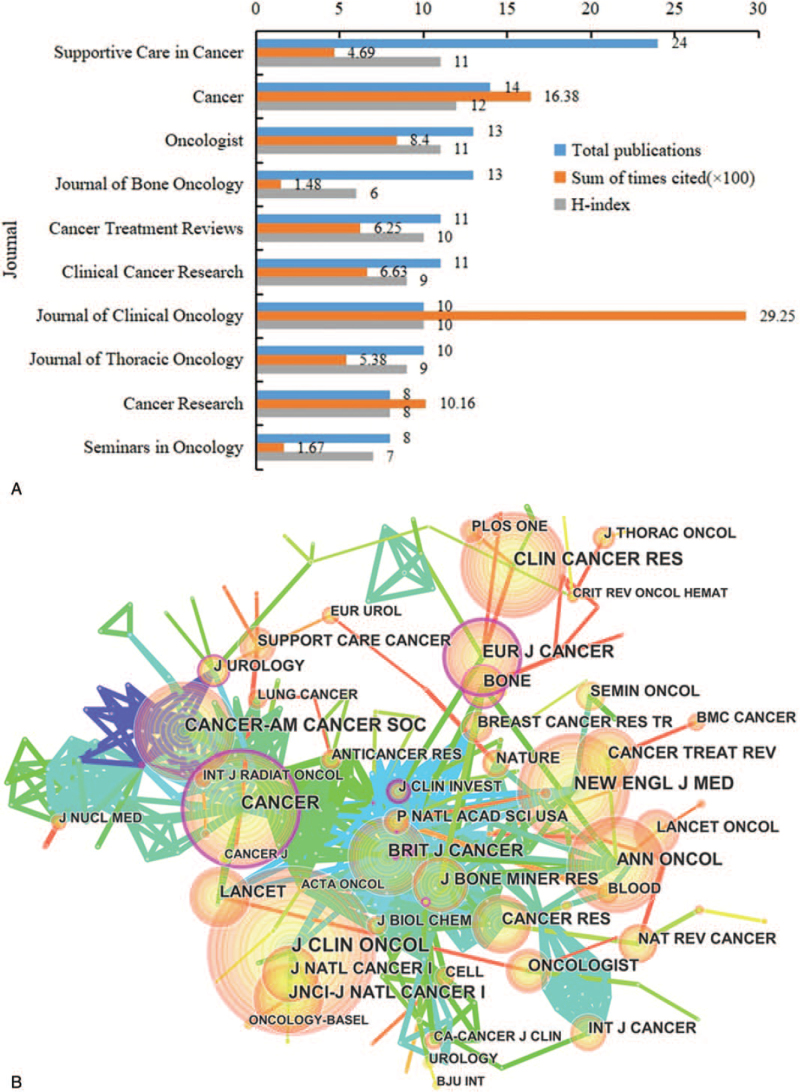
(A) The publication number, H-index, and cited times of the top 10 journals; (B) the journals co-authorship network of publications.

All studies were published in 303 journals. Only 8 (2.6%) journals published more than 10 papers. The journal with the greatest number of publications was *Supportive Care in Cancer,* with a total of 24 (3.5%) papers. At the second position was *Cancer Journal* with 14 (2.05%) papers, followed by *Journal of Oncologist* and *Journal of Bone Oncology* with 13 (1.9%), and *Cancer Treatment Reviews* with 11 (1.6%). The 5 journals account for 10.99% of all the papers. The cited time of *Journal of Clinical Oncology* ranked first (2925), followed by *Cancer* (1638) and *Cancer Research* (1016). The H-index of *Cancer* ranked first (12), followed by *Oncologist* (11) and *Supportive Care in Cancer* (11) (Fig. 3A). Only journals cited a minimum of 50 times were included. *Cancer*, *Eur J Cancer*, and *Cancer-AM* were at the center of research, cooperation between journals is relatively weak (Fig. 3B).

### 3.4 The distribution and co-authorship analysis of institutes

**Figure 4:**
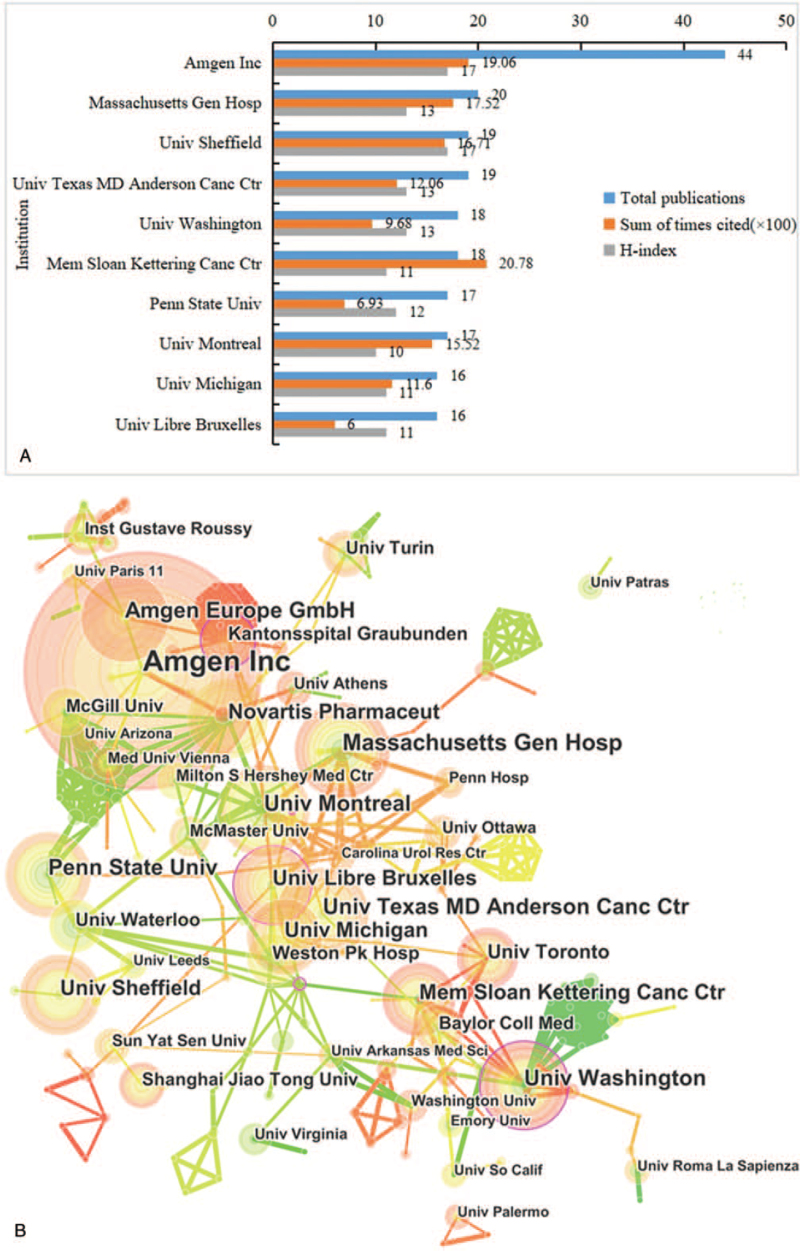
(A) The publication number, H-index, and cited times of the top 10 institutes; (B) the institutes co-authorship network of publications.

The top 10 most productive institutions in the BM field are summarized in Figure 4A. *Amgen Inc.* published the largest number of articles (44), followed by *Massachusetts Gen Hospital* (20), *University Sheffield* (19), *University Texas MD Anderson Cancer Center* (19), and *University Washington* (18). The H-index of cited time of *Amgen Inc.* and *University Sheffield* ranked first (17), followed by *University Washington* (13), *Massachusetts Gen Hospital* (13), and *University Texas MD Anderson Cancer Center* (13). The cited time of *Mem Sloan Kettering Cancer Center* ranked first of all the institutions (2078), follow by *Amgen Inc.* (1906) and *Massachusetts Gen Hospital* (1752). Only institutes with a minimum of 5 articles were included in co-authorship analysis. It shows that *Amgen Inc.*, *Massachusetts Gen Hospital*, and *Washington University* were at the center of researches. In general, cooperation between the institutions were relatively strong (Fig. 4B).

### 3.5 The distribution and co-authorship analysis of authors

**Figure 5:**
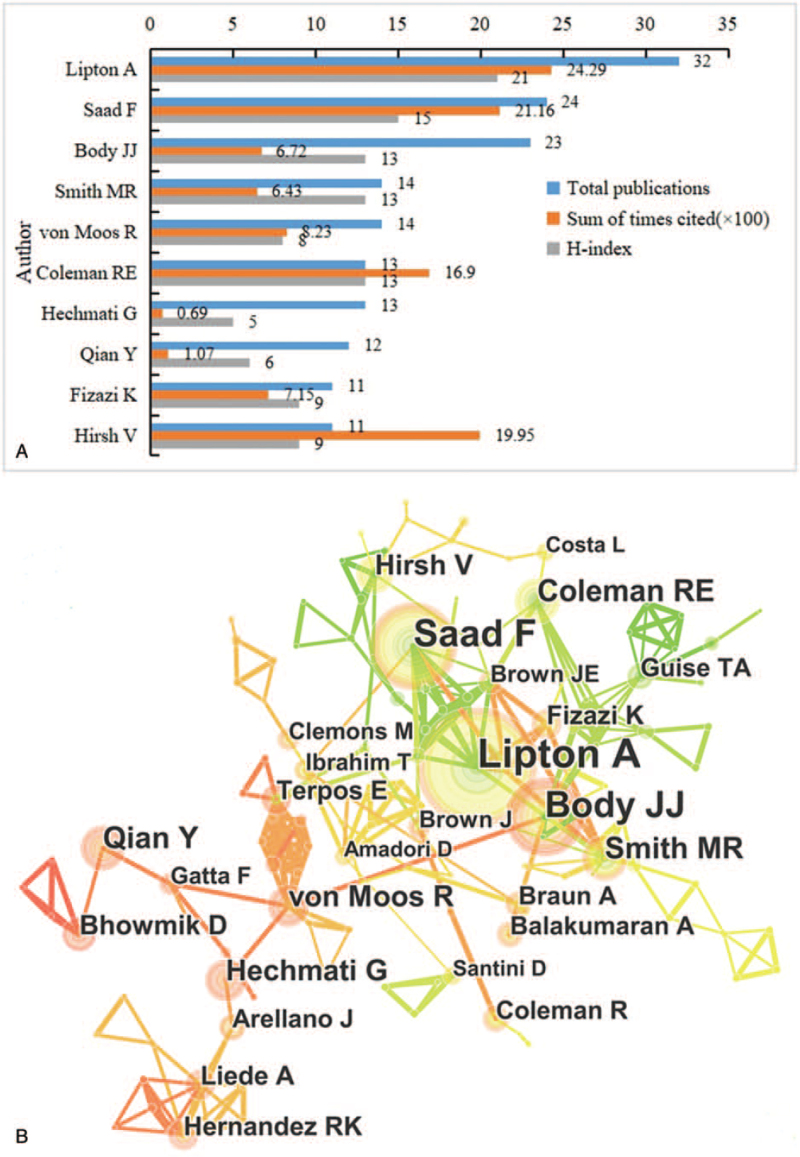
(A) The publication number, H-index, and cited times of the top 10 authors; (B) the authors co-authorship network of publications.

The top 10 most productive authors in the bone metastases field are shown in Figure 5A. *Lipton A* published the largest number of articles (32), follow by *Saad F* (24), *Body JJ* (23), *Smith MR* (14), and *von Moos R* (14). The cited time of *Lipton A* ranked first (2429), followed by *Saad F* (2116) and *Hirsh V* (1995). The H-index of *Lipton A* (21) ranked first of all the authors, followed by *Saad F* (15) and *Body JJ* (13). Only authors published a minimum of 5 articles were included. It shows that authors in the same country have relatively close collaboration. Nevertheless, cooperation between authors from different country is weak (Fig. 5B).

### 3.6 The keywords analysis of research hotspots on study

**Figure 6:**
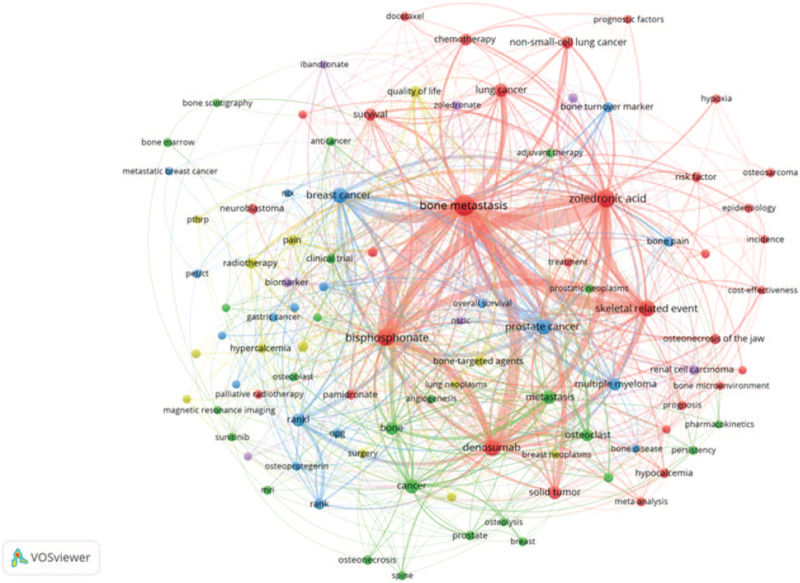
Keywords co-occurrence network of publications.

**Figure 7:**
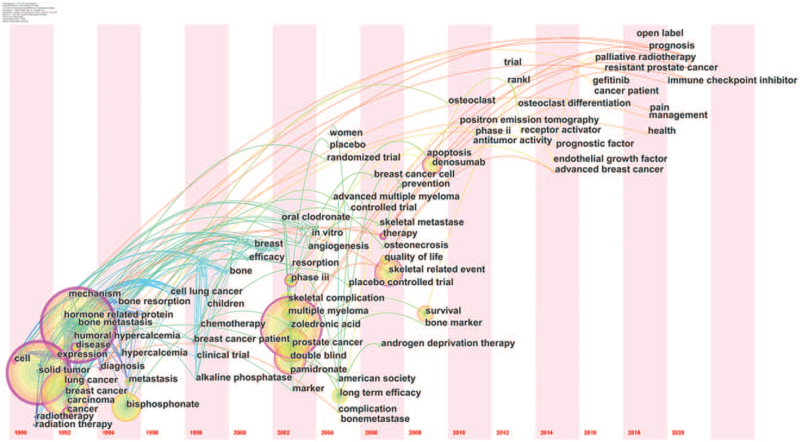
The keywords timeline view of publications.

Keyword co-occurrence can effectively reflect the research hotspots and provide support for the research. In Figure 6, we can see the keywords and research focuses visualized by VOS viewer. The bigger nodes and darker color show a larger weight of the keyword. In addition to “bone metastasis”, “zoledronic acid”, and “skeletal related event” the other core keywords were scattered and link strength was relatively weak. Table 1 shows the 42 meaningful keywords with the strongest citation burst, which represented the research frontiers. The red and blue bars represented the frequently- and infrequently-cited keywords, respectively. Figure 7 shows the keywords timeline view of publications. From this result we can know “Safety”, “skeletal related event”, “open label”, “management”, “health”, and “prognosis” are the research hotspots in the recent years.

## 4 Discussion

### 4.1 Trends of publications related to BM

Bibliometric analysis has been applied vastly to assess the merits of a specific field as a mature tool.^[8,9]^ In order to understand the state of the art of BM, we have carried out a comprehensive analysis of 40 year through bibliometrics. This study has tried to touch upon the BM research with respect to quantity of publications in each year, collaboration from different countries, published journals, the distribution of authors and institutes, and keywords analysis since 1980 to 2020. The result will be helpful in guiding researchers in selecting relevant topics, finding suitable teams to collaborate with, and research platforms to use for their research. From the result of our study, the number of publications rose stably in recent years, especially from 2008 to 2019. It means that the BM research was developing rapidly and attracted more attention in the global medical field. Our study found that the top 5 productive countries (USA, Italy, Canada, China, and UK) made the majority of publications (80.49%), while the top productive institutions were all from these countries. This result showed that the global research on BM concentrated in North America, West Europe, and East Asia.

The number of citations can reflect the quality of a paper. The H-index is a widely used parameter to quantify and standardize researchers’ scientific impact.^[10]^ USA and Canada contributed most in this field with the highest total publications and H-index. It indicates that USA and Canada were the leader in the quantity and the quality. The most important reasons for USA and Canada's achievement may be the rapid development in medical technology, economy, and academy. In addition, adequate research funds are invested in medical research to increase the quality of researches in USA and Canada. While in the top 10 most productive countries, China had the lowest H-index and cited number, which means China's qualities of publications concerning BM were relatively low. China has an advantage in recruitment of participants with the large population and high prevalence of tumor diseases, however China's research ability was relatively weak comparing with West Europe and North America. On the other hand, the amount of invested government funds was much lower than USA and Canada. Only account for 2% to 3% of the total government funds were invested to the medical research in China.^[11]^ But China still has the significant influence and development potential in the field of BM, with the huge population and rapid development of the country.

In publications number of BM field, *Amgen Inc.* ranked first, as well as the H-index, while *Mem Sloan Kettering Cancer Center* was the most cited institution. It indicated that these organizations dominated studies in the field of BM both in terms of quantity and quality. The journal with the greatest number of publications was *Supportive Care in Cancer*, the H-index of *Cancer* ranked first and *Journal of Clinical Oncology* ranked first in cited time, this reflected that these journals were in the leading position and have the great influence in this field. They were the most attractive choice for authors to submit for publication undoubtedly.

Co-authorship research is a meaningful focal point of bibliometrics. The level of research collaboration reflected the current development status of this field. The network map showed that the centrality and density values of the study are not high and relatively weak interaction between countries, institutions, or authors. Considering this result, it is necessary to develop the global collaboration between countries/regions and institutions and build an international academic net of the BM research.

### 4.2 Studies focused on BM

We can find that the research focus on BM in early years was related to pathogenesis and diagnosis, but skeletal related events (SRE), safety and prognosis are the hotspots in recent years. With the advancement of treatment methods and techniques, the survival time of advanced cancer patient was gradually extended. For instance, advanced lung cancer patients’ median survival time was extended to 1 year.^[12]^ While the patients’ survival benefited, SRE risks also increased.^[13–15]^

BM of malignant tumor can be divided into the following 3 types according to the characteristics of the lesion, osteolytic, osteogenic, and mixed.^[16]^ The risk of SRE in patients with bone metastases dominated by osteolytic lesions was high. The pain caused by SRE (bone pain, pathological fracture, spinal cord compression, etc) seriously affect the patients’ life quality. While controlling the primary disease, actively preventing and treating BM SRE are particularly important.

On the basis of the systemic treatment of the primary disease, multiple department treatment should be taken to BM patients. Formulating individualized comprehensive treatment plans in a planned and reasonable manner can reduce or delay the occurrence of BM complications and SRE, which will improve patient's life quality. On the other hand, studies have suggested that among cancer patients, the total prevalence of psychological distress is 35.1%.^[17]^ Psychotherapy for BM patients is indispensable while antidepressants and anti-anxiety drugs can be used for cancer patients’ anxiety and depression symptoms.^[18–20]^

### 4.3 Strengths and limitations

As far as we know, the current study is the first to analyze the quality and quantity of researches using bibliometric analysis and visualization tool in the field of BM. The systematic literature analysis conducted by us consists of comprehensive statistic from various aspects in BM field. As a result, we can get an objective and exhaustive cognition of the global research in BM. Except these obvious strengths, we still found some weaknesses about this study. First some public and commercially available bibliometric databases were not contained in current study such as Medline, Google Scholar, and Scopus. No bibliometric database is considered superior, and there are wide variations in citations data in each database.^[21,22]^ Second, the main evaluation index in this study is citation number and H-index, but there are many factors that affect citation rates.^[23]^

For example, this strategy may favor older articles. Journal and author self-citations, incomplete citing, and omission bias also significantly contribute to citation rates.^[24,25]^ As for H-index, many scholars think that there is an obvious positive correlation between the H-index and journal or region's total publish amount. According to the definition of H-index, it cannot exceed the limit of publication. As a result, it is not conducive to the regions, journals, or authors with small publications and large citation number.

## 5 Conclusion

In this study, we conduct a systematic and comprehensive analysis on the research in BM field. The publication number was rising in recent years stably. USA contributed mostly not only in quality, but also in quantity. *Amgen Inc.* published the largest number of articles, *Supportive Care in Cancer* was the most productive journal related to BM. Safety, skeletal related event, open label, management, health, and prognosis are the research hotspots in recent years. We believe this study can not only show the global research overview in past 40 years but also point the research trend of BM in the future.

## Author contributions

**Conceptualization:** Kai Huang.

**Data curation:** Kai Huang.

**Methodology:** Kai Huang.

**Project administration:** Kai Huang.
